# Supplementary material for: Synthetic protein-binding DNA sponge as a tool to tune gene expression and mitigate protein toxicity
Source: Nat Commun. 2020 Nov 24;11:5961. doi: 10.1038/s41467-020-19552-9 (PMC7686491; doi:10.1038/s41467-020-19552-9)
Supplement: Supplementary file 1 — Supplementary Information [file 41467_2020_19552_MOESM1_ESM.pdf]

Supporting Information for

"Synthetic protein-binding DNA sponge as a tool to tune gene expression and mitigate protein toxicity"

Xinyi Wan, Filipe Pinto, Luyang Yu and Baojun Wang

Supplementary Figures

Supplementary Fig. 1: Plasmid maps of the ligand responsive circuits used in this study. 2

Supplementary Fig. 2: Plasmid maps of the DNA sponges used in this study. 3

Supplementary Fig. 3: Synthetic DNA sponge enables tuning circuit's output expression by decoying receptors (TetR or LuxR) within the aTc or AHL-responsive single-layered circuit.......................... 4

Supplementary Fig. 4: Comparison of circuit output responses using sponges containing tetO and P_{tet2} repeats to decoy TetR within the aTc-responsive single-layered circuit. 5

Supplementary Fig. 5: Single cell assay of the dose-responses of the aTc-responsive single-layered circuit under regulation by the tetO- or P_{tet2}-based sponges. 6

Supplementary Fig. 6: Single cell assay of the dose-response of the AHL-responsive single-layered circuit under regulation by the LBS-containing sponges. 7

Supplementary Fig. 7: P_{tet2}-based sponge tunes circuit's output gene expression by decoying TetR within the two-layered circuit's signal processing module. 8

Supplementary Fig. 8: Comparison of circuit output responses using sponges containing tetO or P_{tet2} by decoying TetR within the two-layered circuit's signal processing module. 9

Supplementary Fig. 9: Single cell assay of a two-layered AHL-responsive circuit's dose responses under regulation by the tetO- and P_{tet2}-based sponges. 10

Supplementary Fig. 10: P_{ecf11}-based sponge tunes circuit's output gene expression and cellular burden by decoying ECF11 within a two-layered circuit's signal processing module. 11

Supplementary Fig. 11: Tuning response of a two-layered circuit using dual-layer DNA sponges to decoy regulators within both the input sensing and signal processing modules. 13

Supplementary Fig. 12: Dual-layer DNA sponges provide additive tuning and mitigation effects on multi-layered circuit's response and burden on the host. 14

Supplementary Fig. 13: Stability assay of synthetic DNA sponges. 16

Supplementary Fig. 14: Gating strategy used in flow cytometry assays. 17

Supplementary Data (supplied as individual excel data files)

Supplementary Data 1: List of constructs and sequences used in this study. 18

Supplementary Data 2: Best model fits used in this study. 18

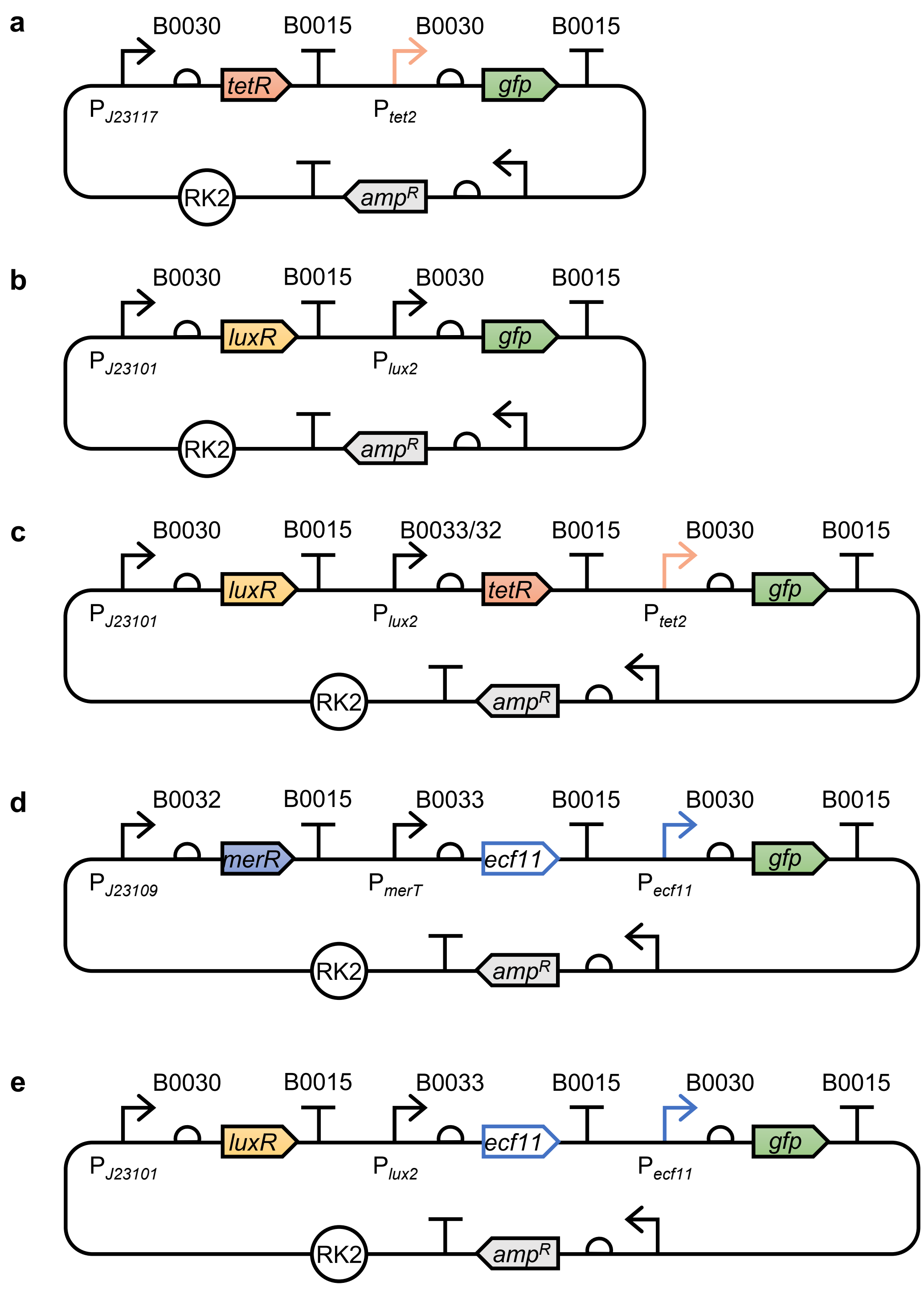

Supplementary Fig. 1: Plasmid maps of the ligand responsive circuits used in this study. **a**, aTc responsive single-layered circuit, related to **Fig. 2a**. **b**, Quorum sensing molecule ($3OC_6HSL$) responsive single-layered circuit, related to **Fig. 2f**. **c**, Two-layered AHL-responsive circuit containing a TetR-based inverter, related to **Figs. 3a** (with B0033-TetR)**, 4a** (with B0032-TetR). **d**, Two-layered mercury-responsive circuit containing an ECF11-based transcriptional amplifier, related to **Fig. 3e**. **e**, Two-layered AHL-responsive circuit containing an ECF11-based transcriptional amplifier, related to **Fig. 5a**.

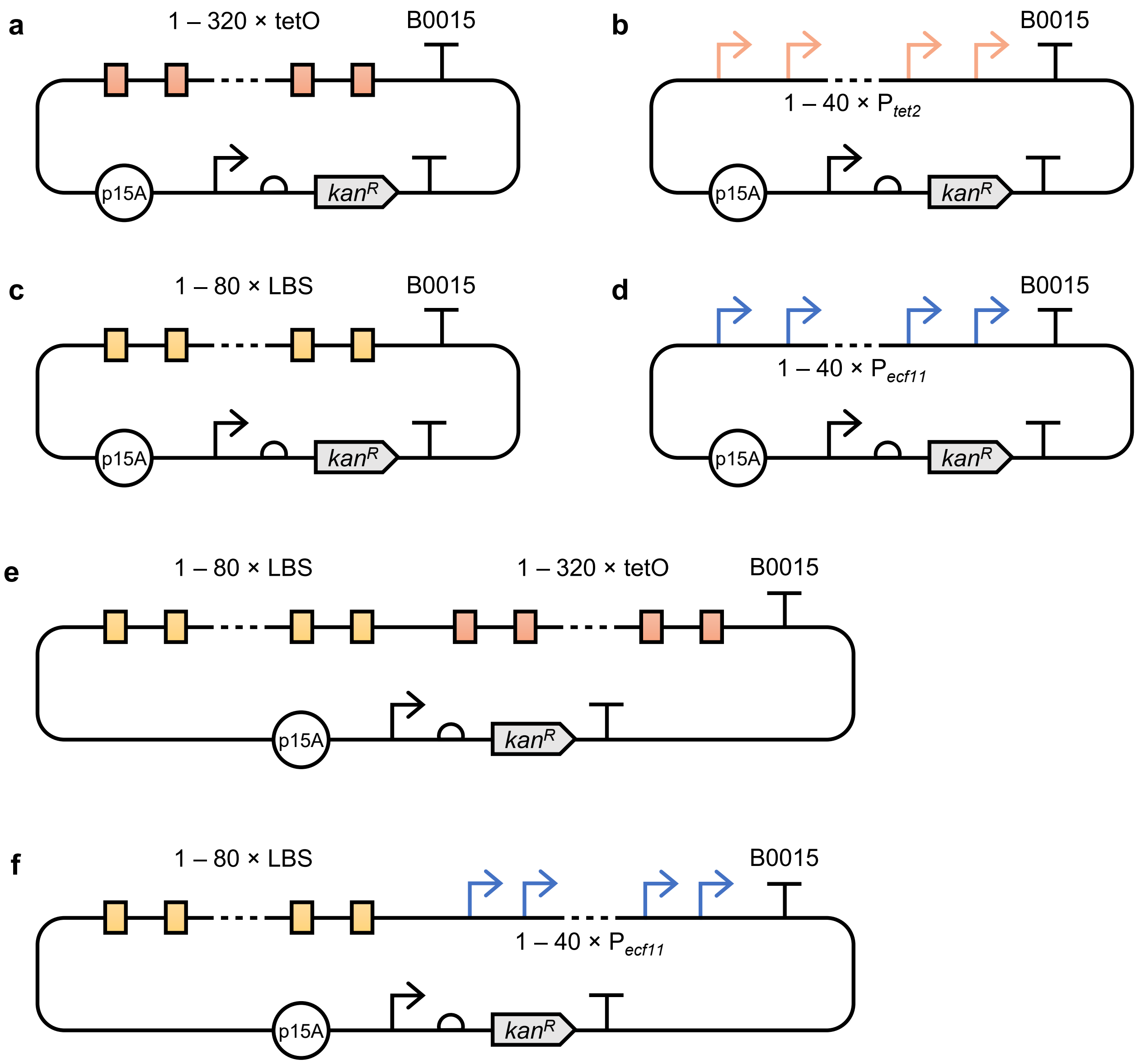

Supplementary Fig. 2: Plasmid maps of the DNA sponges used in this study.

a, Sponge plasmid containing 1 to 320 repeats of tetO site, related to **Figs. 2a, 3a, 4a**, **Supplementary Fig. 3a**. **b**, Sponge plasmid containing 1 to 40 repeats of P_{tet2}, related to **Fig. 2a**, **Supplementary Figs. 3a, 7a**. **c**, Sponge plasmid containing 1 to 80 repeats of LuxR binding site (LBS), related to **Fig. 2f, 4a, 5a**, **Supplementary Fig. 3g**. **d**, Sponge plasmid containing 1 to 40 repeats of P_{ecf11}, related to **Fig. 3e, 5a**. **e**, LBS-tetO dual-layer sponge plasmid containing 1 to 80 repeats of LBS and 1 to 320 repeats of tetO site, related to **Fig. 4a**. **f**, LBS-P_{ecf11} dual-layer sponge plasmid containing 1 to 80 repeats of LBS and 1 to 40 repeats of P_{ecf11}, related to **Fig. 5a**.

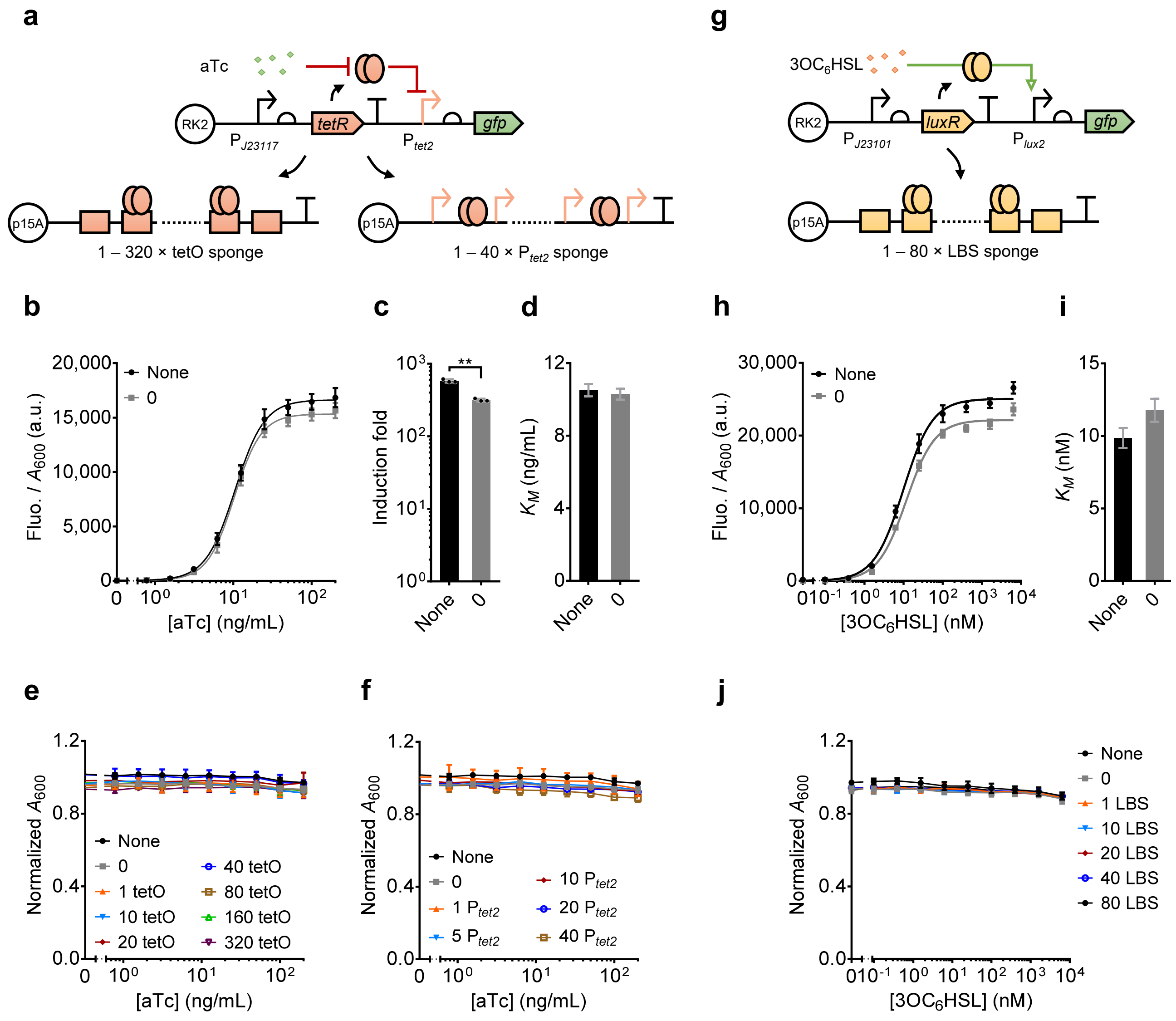

Supplementary Fig. 3: Synthetic DNA sponge enables tuning circuit's output expression by decoying receptors (TetR or LuxR) within the aTc or AHL-responsive single-layered circuit. **a**, Schematic of the design of two types of DNA sponges to decoy TetR in the aTc-responsive circuit: 1–320 repeats of TetR operator (tetO) or 1–40 repeats of P_{tet2} promoter. **b−d**, The aTc-responsive circuit's dose responses (**b**), induction fold (**c**) and Hill constant (K_M) of the circuit's fitted dose responses (**d**) with (0) or without (None) the empty sponge plasmid. Induction fold was calculated between uninduced and 200 ng/mL aTc induced samples of the circuit. Statistical difference was determined by a two-tailed Welch's *t* test: $p = 0.0011$, t = 13.7. **e,f**, Normalized cell densities of circuits tested in **Fig. 2b,c**. **g**, Design of the AHL ($3OC_6HSL$) responsive circuit with sponges containing 1–80 repeats of LuxR binding site (LBS). **h,i**, Dose response (**h**) of the AHL-responsive circuit with (0) or without (None) empty sponge plasmid and the Hill constant (K_M) of the circuit's fitted dose responses (**i**). **j**, Normalized cell densities of circuits tested in **Fig. 2g**. Cell densities were normalized to the negative control (carrying empty circuit and empty sponge plasmids). For **b,c,e,f,h** and **j**, values are mean ± s.d. (*n* = 3 biologically independent samples). For **d** and **i**, values are mean ± s.e.m. (*n* = 3 biologically independent samples). Fluo., fluorescence. a.u., arbitrary units. *p* value summary: *****p* value < 0.0001, 0.0001 < ****p* value < 0.001, 0.001 < ***p* value < 0.01, 0.01 < **p* value < 0.05, *p* > 0.05: n.s.

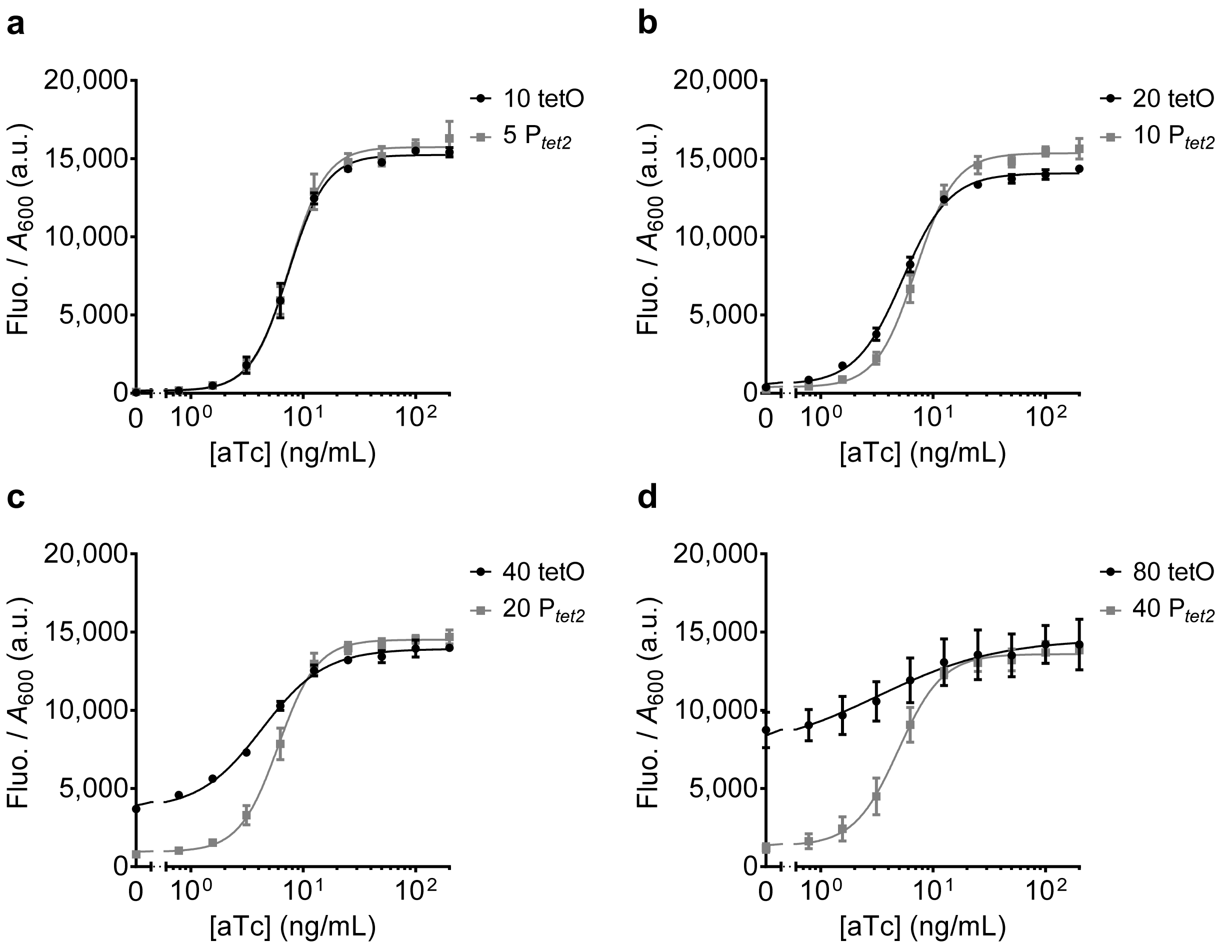

Supplementary Fig. 4: Comparison of circuit output responses using sponges containing tetO and P_{tet2} repeats to decoy TetR within the aTc-responsive single-layered circuit. The effects of the tetO- or P_{tet2}-based sponge containing the same number of repeats of TetR binding site on the circuit output expression (**Fig. 2a**) were compared under the same induction and incubation condition. Each tetO is a TetR binding site, and each P_{tet2} has two tetO. Data were collected at 5 h post induction and incubation. Values are mean ± s.d. (n = 3 biologically independent samples). Fluo., fluorescence. a.u., arbitrary units.

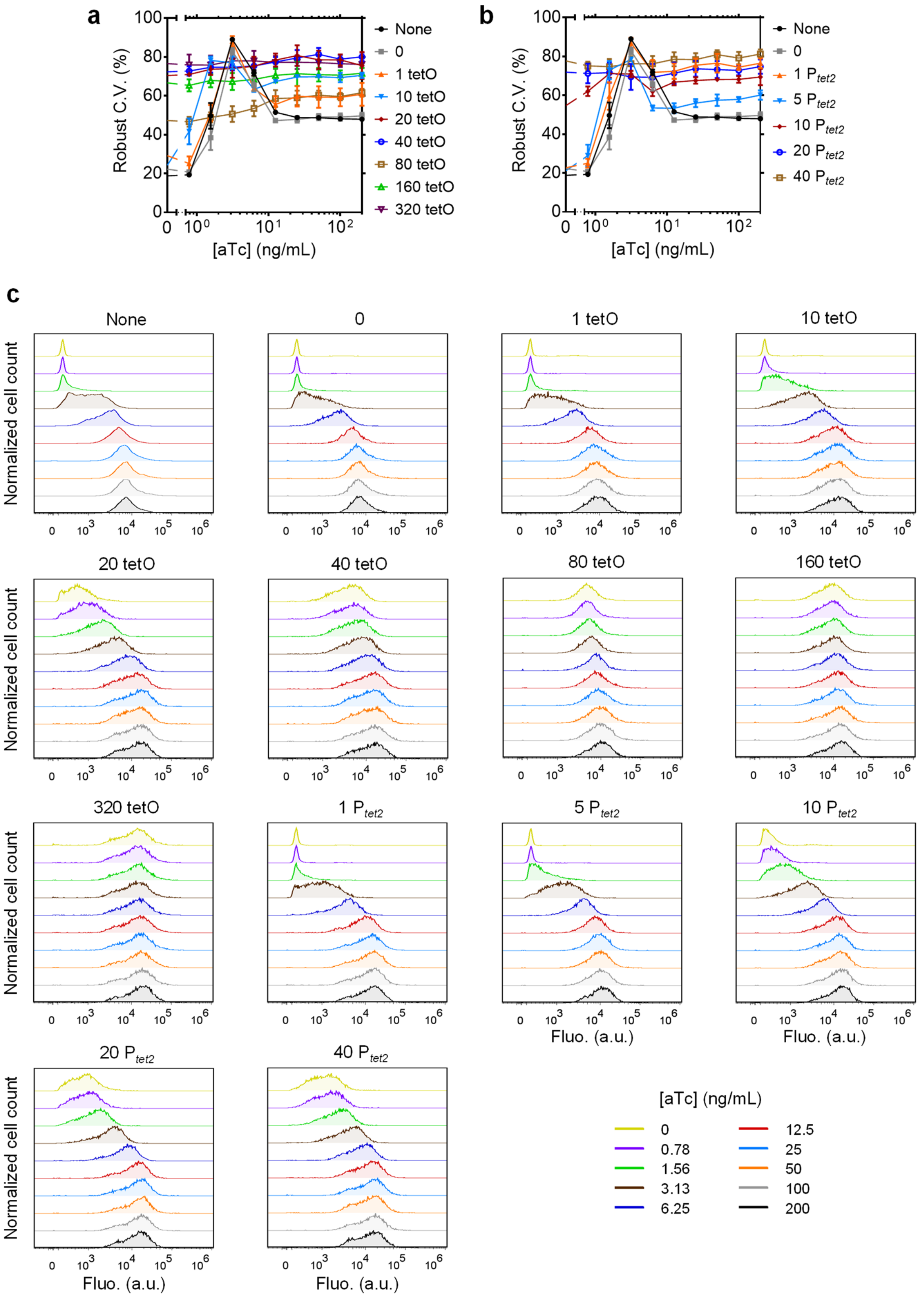

Supplementary Fig. 5: Single cell assay of the dose-responses of the aTc-responsive single-layered circuit under regulation by the tetO- or P_{tet2}-based sponges.

a,**b**, Robust C.V. (coefficient of variation) of the output gene expression of the aTc-responsive circuit (**Fig. 2a**, **Supplementary Fig. 3a**) with sponges containing 1–320 repeats of tetO (**a**) or 1–40 repeats of P_{tet2} (**b**) from single cell assay. Values are mean ± s.d. (n = 3 biologically independent samples). **c**, Dose responses of the circuit with the tetO- or P_{tet2}-based sponge at single cell level under different aTc inductions. Fluo., fluorescence. a.u., arbitrary units.

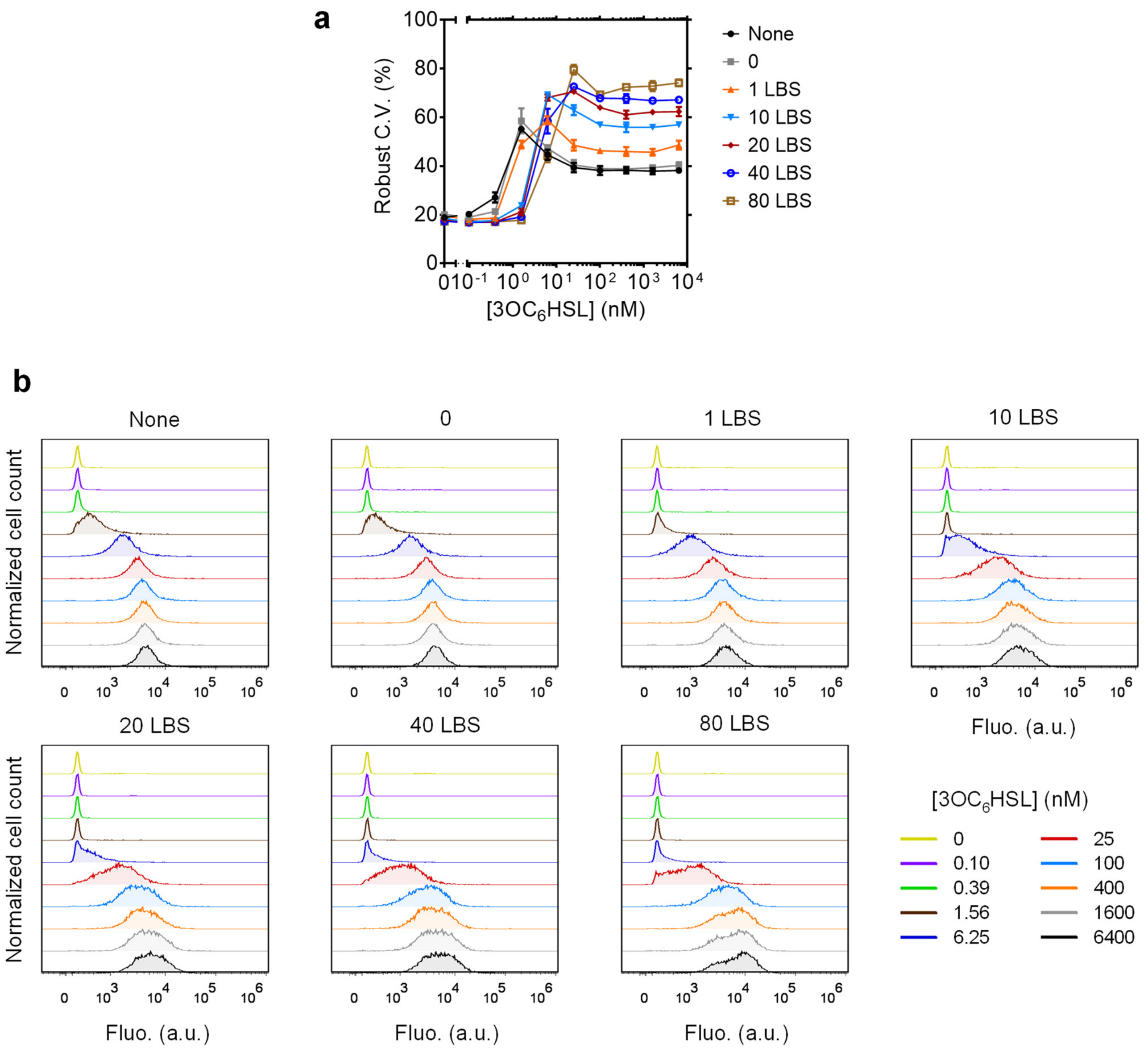

Supplementary Fig. 6: Single cell assay of the dose-response of the AHL-responsive single-layered circuit under regulation by the LBS-containing sponges.

a, Robust C.V. (coefficient of variation) of the output gene expression of the AHL-responsive circuit (**Fig. 2f**) with sponges containing 1–80 repeats of LuxR binding site (LBS) from single cell assay. Values are mean ± s.d. (n = 3 biologically independent samples). **b**, Dose responses of the circuit with the LBS-containing sponge at single cell level under different 3OC$_6$HSL inductions. Fluo., fluorescence. a.u., arbitrary units.

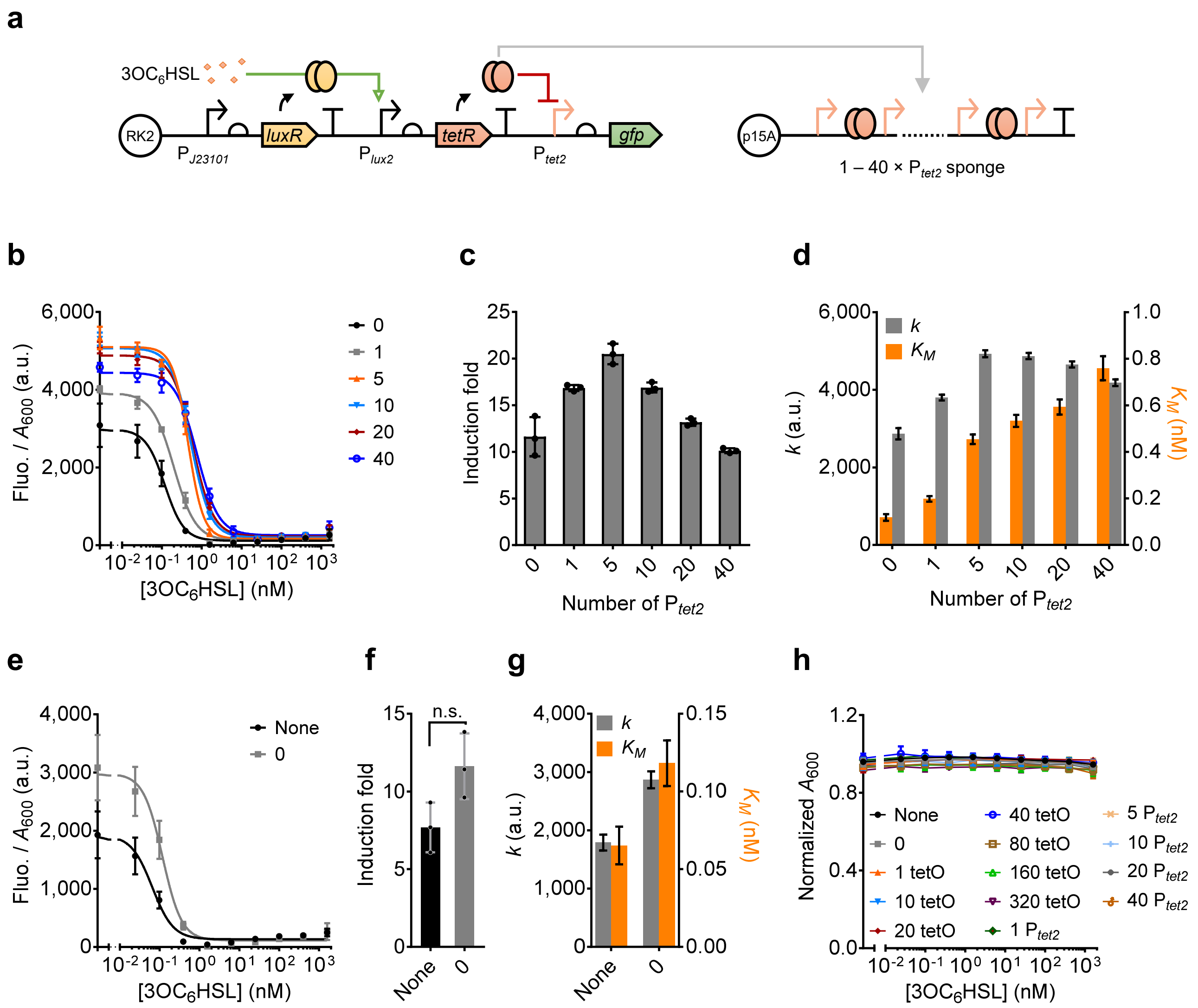

Supplementary Fig. 7: P_{tet2}-based sponge tunes circuit's output gene expression by decoying TetR within the two-layered circuit's signal processing module.

a, Schematic of a two-layered AHL-responsive circuit regulated by DNA sponges containing 1–40 P_{tet2} repeats. **b**, Characterization of the circuit in response to various concentrations of $3OC_6HSL$ and with sponges containing 1–40 P_{tet2} repeats. **c**, Induction fold between uninduced and 1.6 μM $3OC_6HSL$ induced samples of the circuit characterized in **b**. **d**, Hill constant (K_M) and maximum output (k) of the fitted dose responses of the circuit characterized in **b**. **e,f**, Dose responses (**e**) and induction fold (**f**) of the circuit with (0) or without (None) the empty sponge plasmid. The induction fold was calculated between uninduced and 1.6 μM $3OC_6HSL$ induced samples of the circuit characterized in **e**. Statistical difference was determined by a two-tailed Welch's t test: $p = 0.0663$, t = 2.572. **g**, Hill constant (K_M) and maximum output (k) of the fitted dose responses of the circuit with (0) or without (None) the empty sponge plasmid. **h**, Normalized cell densities of the circuits tested in **b** and **Fig. 3b**. Cell densities were normalized to the negative control (carrying empty circuit and empty sponge plasmids). For **b,c,e,f** and **h**, values are mean ± s.d. (n = 3 biologically independent samples). For **d** and **g**, values are mean ± s.e.m. (n = 3 biologically independent samples). Fluo., fluorescence. a.u., arbitrary units. p value summary: ****p value < 0.0001, 0.0001 < ***p value < 0.001, 0.001 < **p value < 0.01, 0.01 < *p value < 0.05, p > 0.05: n.s.

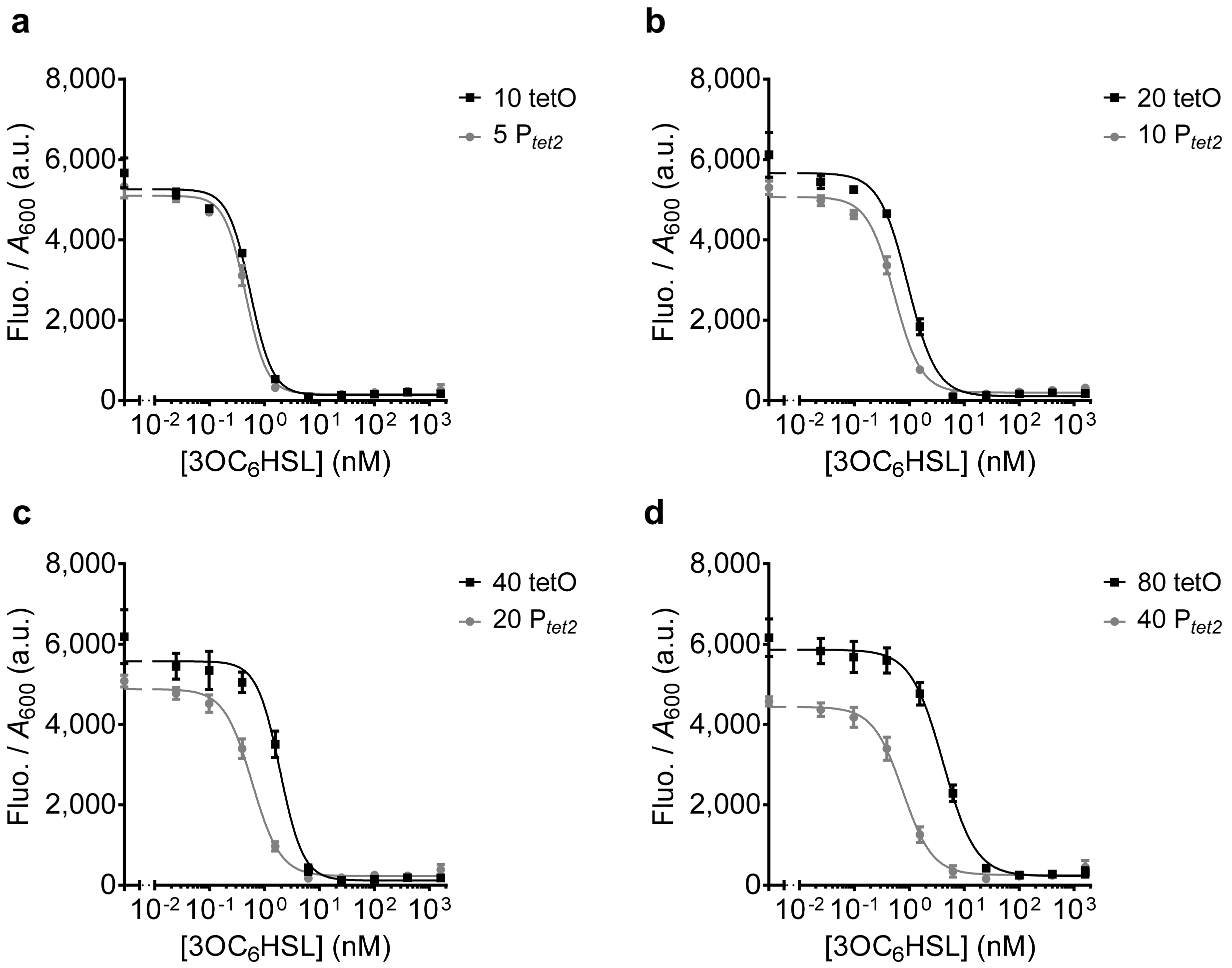

Supplementary Fig. 8: Comparison of circuit output responses using sponges containing tetO or P_{tet2} by decoying TetR within the two-layered circuit's signal processing module. The effects of the tetO- or P_{tet2}-based sponge containing the same number of TetR binding sites on the output expression of a two-layered AHL-responsive circuit (**Fig. 3a**, **Supplementary Fig. 7a**) were compared under the same induction and incubation condition. Each tetO is a TetR binding site, and each P_{tet2} has two tetO. Data were collected at 5 h post induction and incubation. Values are mean ± s.d. (*n* = 3 biologically independent samples). Fluo., fluorescence. a.u., arbitrary units.

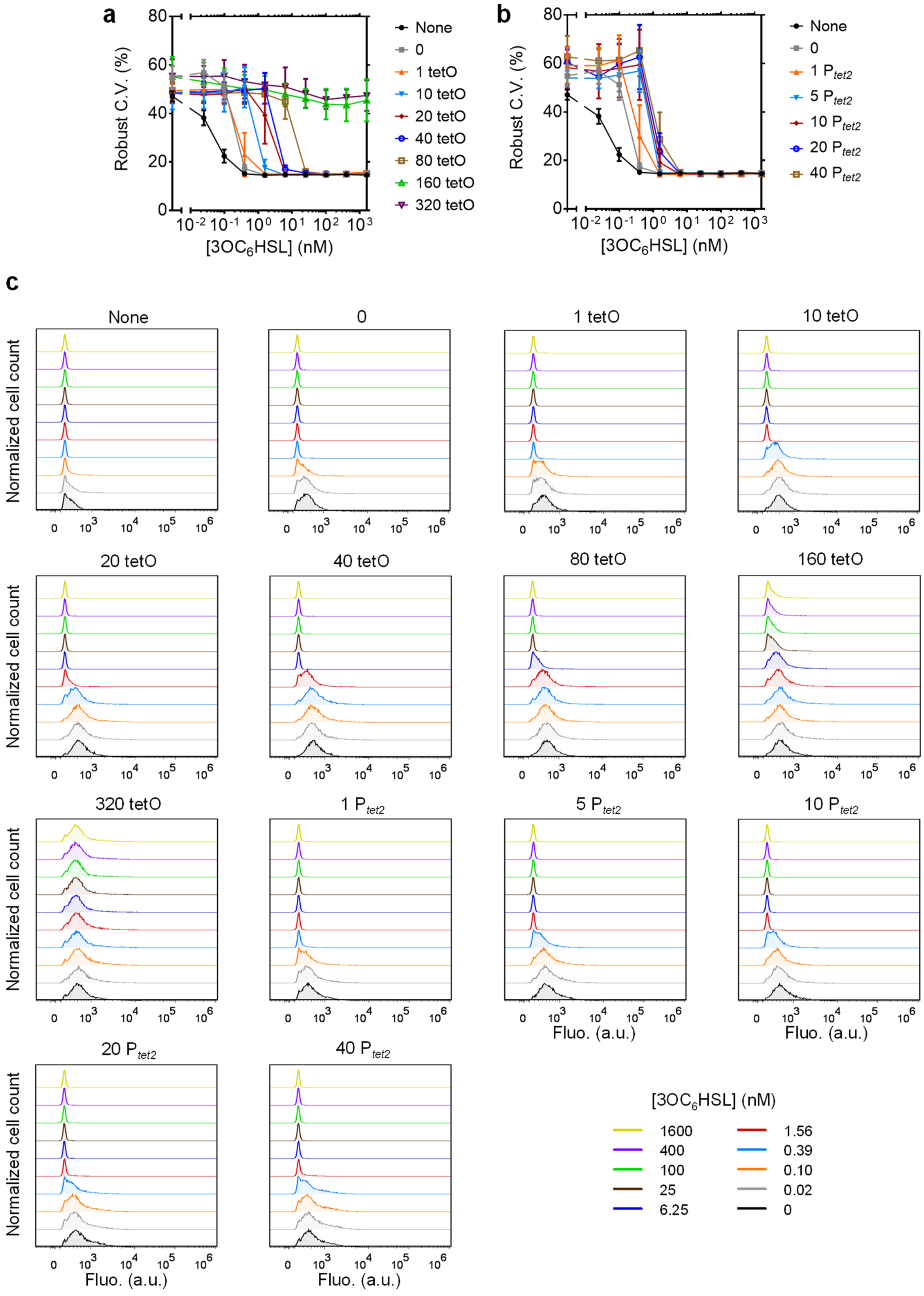

Supplementary Fig. 9: Single cell assay of a two-layered AHL-responsive circuit's dose responses under regulation by the tetO- and P_{tet2}-based sponges.

a,**b**, Robust C.V. (coefficient of variation) of the output gene expression of the AHL-responsive two-layered circuit (**Fig. 3a**, **Supplementary Fig. 7a**) with sponges containing 1–320 tetO repeats (**a**) or 1–40 P_{tet2} repeats (**b**) from single cell assay. Values are mean ± s.d. (*n* = 3 biologically independent samples). **c**, Dose responses of the circuit with the tetO- or P_{tet2}-based sponges at single cell level under different $3OC_6HSL$ inductions. Fluo., fluorescence. a.u., arbitrary units.

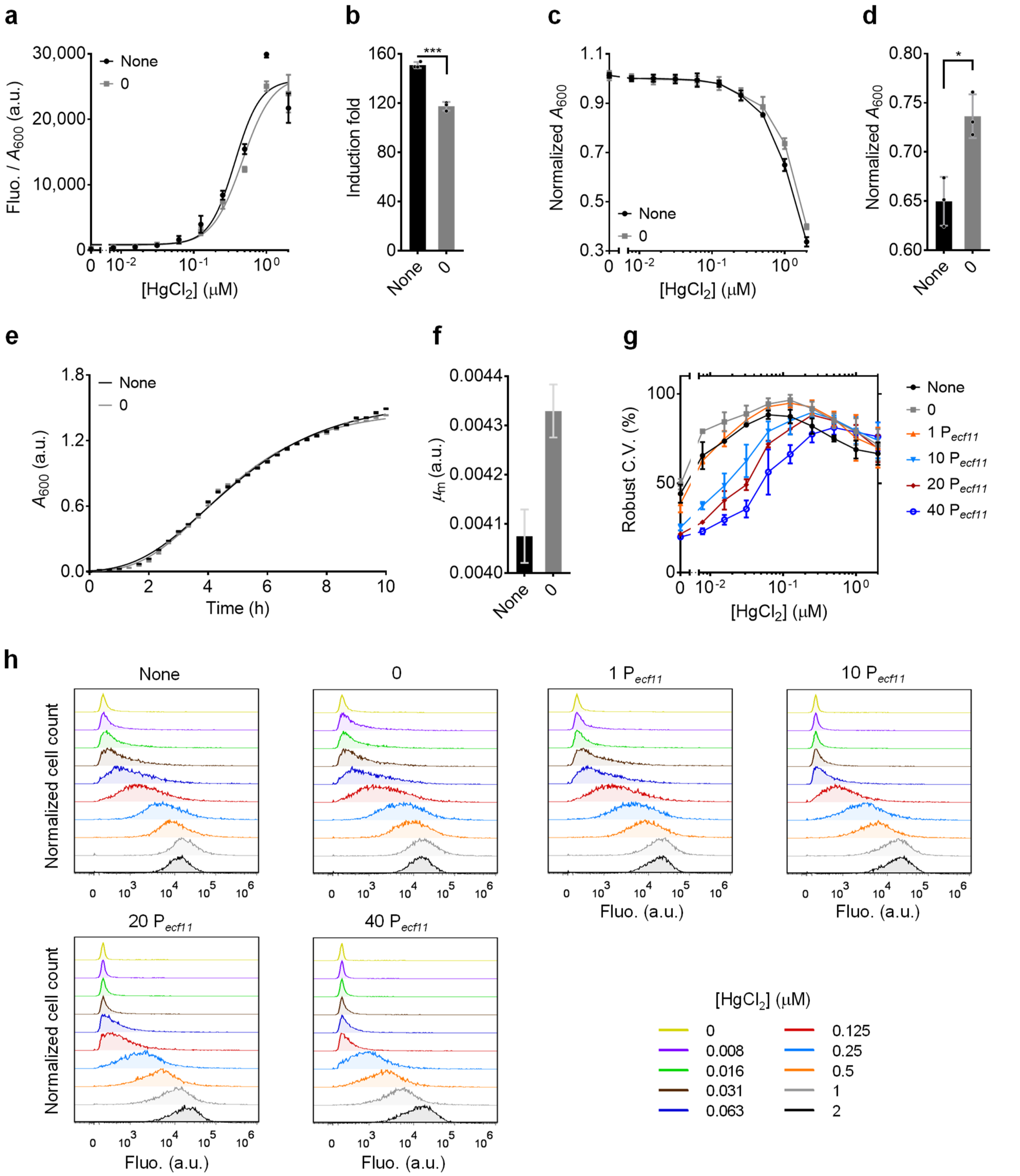

Supplementary Fig. 10: P$_{ecf11}$-based sponge tunes circuit's output gene expression and cellular burden by decoying ECF11 within a two-layered circuit's signal processing module. **a**, Characterization of a two-layered mercury-responsive circuit (**Fig. 3e**) in response to various concentrations of $HgCl_2$ with (0) or without (None) the empty sponge plasmid. **b**, Induction fold between uninduced and 1 μM $HgCl_2$ induced samples of the circuit characterized in **a**. **c**, Normalized cell densities of the circuit tested in **a**. The cell densities were normalized to the negative control (carrying empty circuit and empty sponge plasmids). **d**, Bar chart showing normalized cell densities of the circuit induced with 1 μM mercury as characterized in **c**. **e**, Gompertz model fitted cell growth curves of the circuit with (0) or without (None) the empty sponge plasmid in response to 1 μM mercury. **f**, Growth rate (μ_m) of the cells at exponential growth phase

as characterized in **e**. **g**, Robust C.V. (coefficient of variation) of the circuit's output gene expression with sponges containing 1–40 P_{ecf11} repeats from single cell assay. **h**, Dose responses of the circuit with the P_{ecf11}-containing sponge at single cell level under different $HgCl_2$ inductions. For **a**–**e** and **g**, values are mean ± s.d. (n = 3 biologically independent samples). For **f**, values are mean ± s.e.m. (n = 3 biologically independent samples). Statistical difference was determined by a two-tailed Welch's *t* test: **b**, $p = 0.0003$, t = 13.48; **d**, $p = 0.0111$, t = 4.502. Fluo., fluorescence. a.u., arbitrary units. *p* value summary: ****p value < 0.0001, 0.0001 < ***p value < 0.001, 0.001 < **p value < 0.01, 0.01 < *p value < 0.05, $p > 0.05$: n.s.

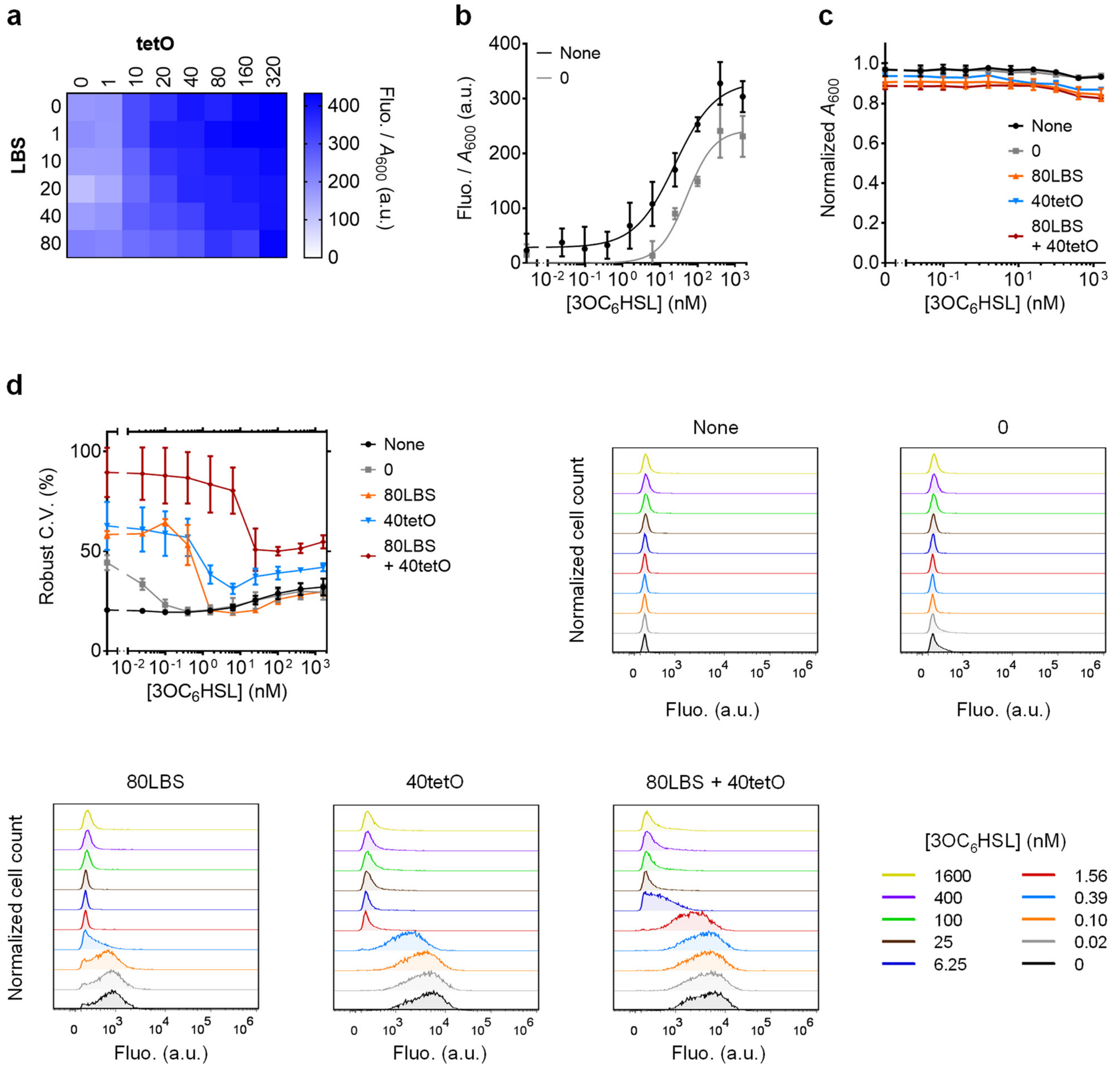

Supplementary Fig. 11: Tuning response of a two-layered circuit using dual-layer DNA sponges to decoy regulators within both the input sensing and signal processing modules. **a**, Output gene expression of a two-layered AHL-responsive circuit (**Fig. 4a**) with LBS-tetO dual-layer sponges under 1.6 μM $3OC_6HSL$ induction. **b**, Dose response of the circuit with (0) or without (None) the empty dual-layer sponge plasmid. **c**, Normalized cell densities of the circuit with four different LBS-P_{ecf11} sponges as tested in **Fig. 4b**. **d**, Robust C.V. (coefficient of variation) of the output gene expression and dose responses of the circuit with LBS-tetO dual-layer sponges from single cell assay. For **a**, values are mean (n = 3 biologically independent samples). For **b–d**, values are mean ± s.d. (n = 3 biologically independent samples). Fluo., fluorescence. a.u., arbitrary units.

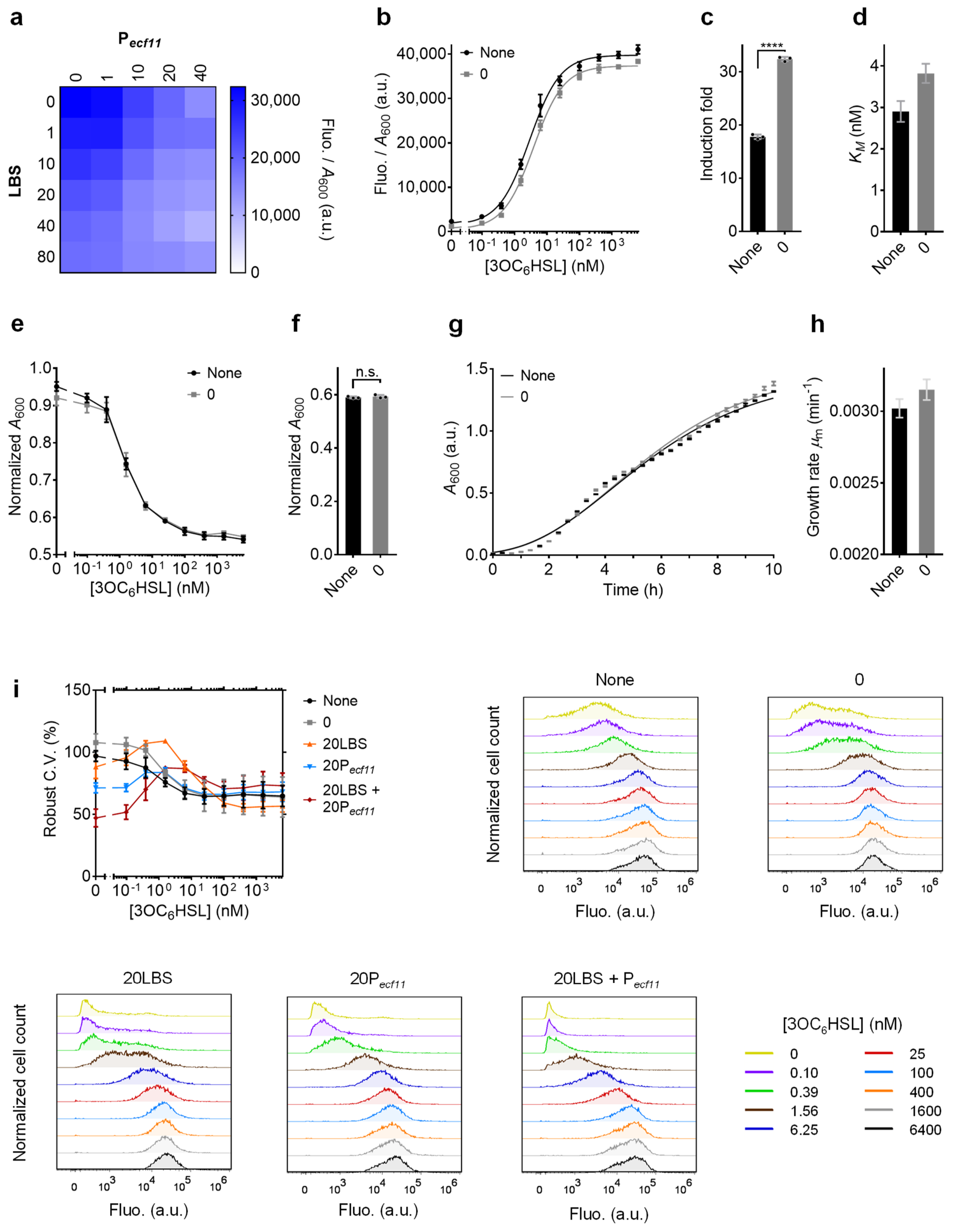

Supplementary Fig. 12: Dual-layer DNA sponges provide additive tuning and mitigation effects on multi-layered circuit's response and burden on the host.

a, Output expression of a two-layered AHL-responsive circuit (**Fig. 5a**) regulated by various LBS-P_{ecf11} dual-layer sponges under 25 nM 3OC$_6$HSL induction. **b,c**, Dose response (**b**) and induction fold (**c**) of the circuit with (0) or without (None) the sponge plasmid. Induction fold was calculated between uninduced and 6.4 μM 3OC$_6$HSL induced samples of the circuit characterized in **b**. **d**, Hill constant (K_M) of the fitted dose responses of the circuit characterized in **b**. **e**, Normalized cell densities of the cell strains tested in **b**. Cell densities were normalized to the negative control

(carrying empty circuit and empty sponge plasmids). **f**, Bar chart showing normalized cell densities of the circuits induced with 25 nM $3OC_6HSL$ as tested in **e**. **g**, Gompertz model fitted cell growth curves of the circuit with (0) or without (None) the sponge plasmid in response to 25 nM $3OC_6HSL$. **h**, Growth rate (μ_m) of the cell strains at exponential growth phase as characterized in **g**. Statistical difference was determined by a two-tailed Welch's *t* test: **c**, $p < 0.0001$, t = 42.63; **f**, $p = 0.4856$, t = 0.7885. **i**, Robust C.V. (coefficient of variation) of the output gene expression and dose responses of the circuit with LBS-P_{ecf11} dual-layer sponges from single cell assay. For **a**, values are mean (n = 3). For **b**,**c**,**e–g** and **i**, values are mean ± s.d. (n = 3 biologically independent samples). For **d** and **h**, values are mean ± s.e.m. (n = 3 biologically independent samples). Fluo., fluorescence. a.u., arbitrary units. *p* value summary: ****p value < 0.0001, 0.0001 < ***p value < 0.001, 0.001 < **p value < 0.01, 0.01 < *p value < 0.05, $p > 0.05$: n.s.

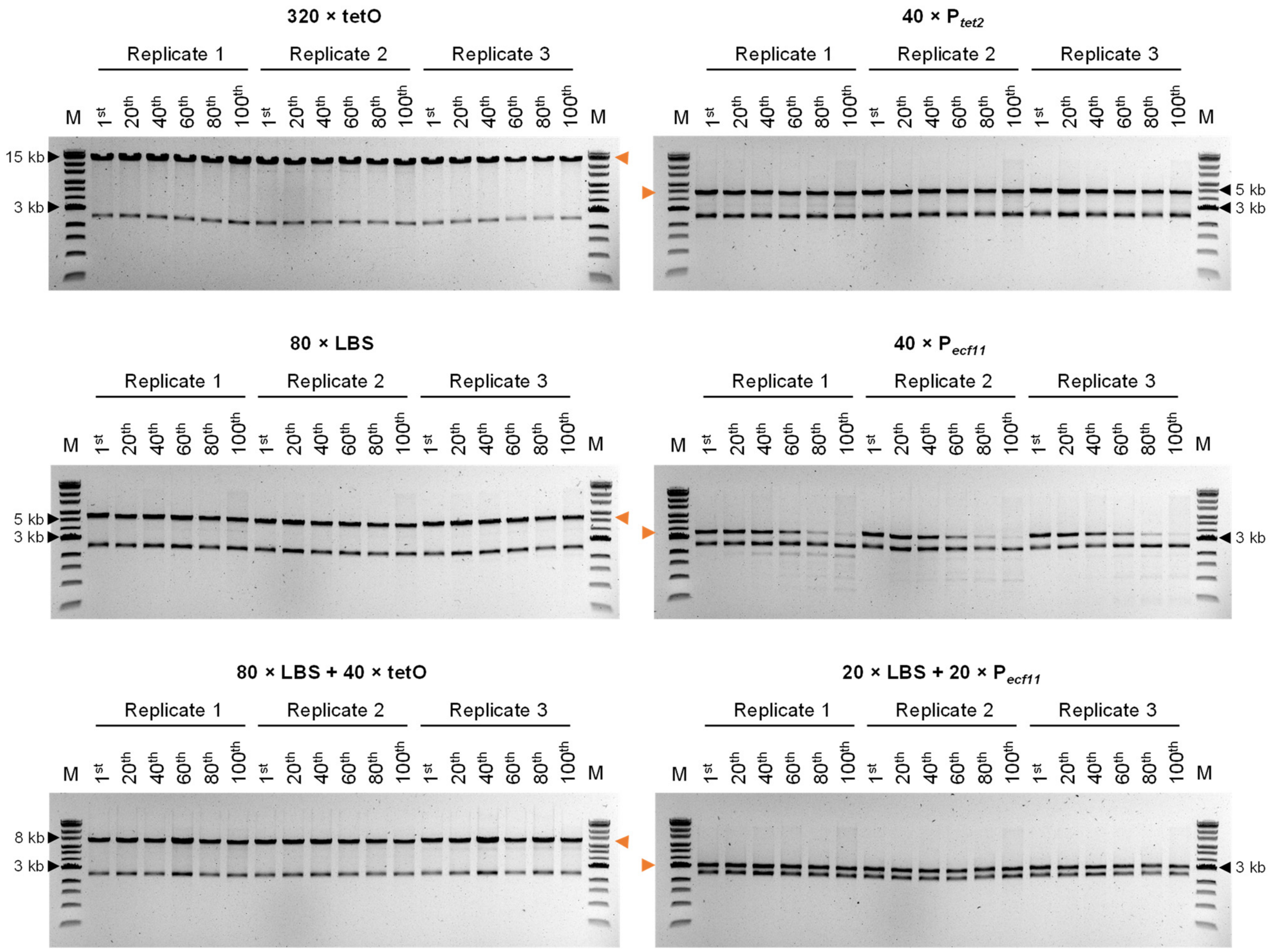

Supplementary Fig. 13: Stability assay of synthetic DNA sponges.

Image of gels post electrophoresis showing the genetic stability of synthetic single-layer DNA sponges of 320 × tetO, 40 × P_{tet2}, 80 × LBS, 40 × P_{ecf11} and dual-layer DNA sponges of 80 × LBS + 40 × tetO and 20 × LBS + 20 × P_{ecf11} respectively. The sponge plasmids were extracted from the host cells after the 1st, 20th, 40th, 60th, 80th and 100th generations before restriction digestion. Orange arrows point to the positions of the digested DNA sponge constructs in each gel image. Three colonies of each sponge were tested as three biological replicates.

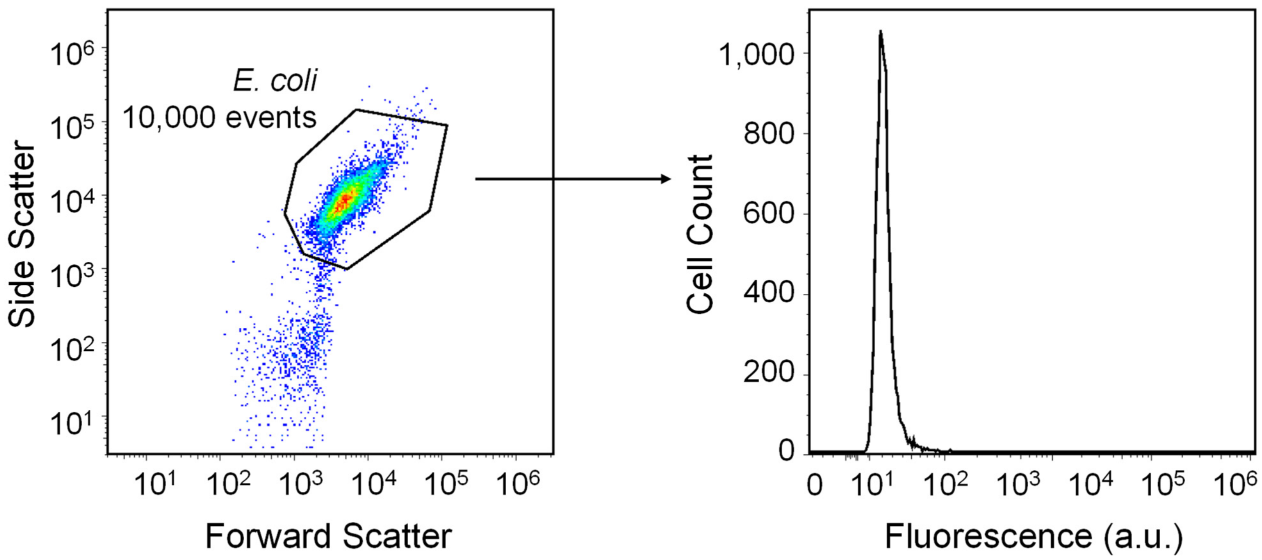

Supplementary Fig. 14: Gating strategy used in flow cytometry assays.

Image exemplifying the gating strategy used for flow cytometry assays in this study. *E. coli* population was selected with an appropriate gate of forward-scattering and side-scattering (left panel).10,000 total *E. coli* cell events were collected for fluorescence analysis (right panel).

Supplementary Data (supplied as individual excel data files)

Supplementary Data 1: List of constructs and sequences used in this study.

Table 1: Plasmids used in this study.

Table 2: List of genetic parts and sequences constructed in this study.

Table 3: List of oligonucleotides used in this study.

Supplementary Data 2: Best model fits used in this study.

Table 1: Best model fits for the characterized dose-responses of the various genetic circuits in this study.

Table 2: Best model fits for the characterized growth curves of the cell strains comprising various circuits in this study.
