# Supplementary material for: Synthetic protein-binding DNA sponge as a tool to tune gene expression and mitigate protein toxicity
Source: Nat Commun. 2020 Nov 24;11:5961. doi: 10.1038/s41467-020-19552-9 (PMC7686491; doi:10.1038/s41467-020-19552-9)
Supplement: Supplementary file 4 — Description of Additional Supplementary Files [file 41467_2020_19552_MOESM4_ESM.pdf]

Title: Supplementary Data 1:

Description: List of constructs and sequences used in this study. Table 1: Plasmids used in this study. Table 2: List of genetic parts and sequences constructed in this study. Table 3: List of oligonucleotides used in this study.

Title: Supplementary Data 2:

Description: Best model fits used in this study. Table 1: Best model fits for the characterized dose-responses of the various genetic circuits in this study. Table 2: Best model fits for the characterized growth curves of the cell strains comprising various circuits in this study
