# Supplementary material for: Synthetic protein-binding DNA sponge as a tool to tune gene expression and mitigate protein toxicity
Source: Nat Commun. 2020 Nov 24;11:5961. doi: 10.1038/s41467-020-19552-9 (PMC7686491; doi:10.1038/s41467-020-19552-9)
Supplement: Supplementary file 6 — Reporting Summary [file 41467_2020_19552_MOESM6_ESM.pdf]

nature research

Corresponding author(s): Baojun Wang

Last updated by author(s): Oct 17, 2020

Reporting Summary

Nature Research wishes to improve the reproducibility of the work that we publish. This form provides structure for consistency and transparency in reporting. For further information on Nature Research policies, see our Editorial Policies and the Editorial Policy Checklist.

Statistics

For all statistical analyses, confirm that the following items are present in the figure legend, table legend, main text, or Methods section.

n/a	Confirmed	
☐	☒	The exact sample size (*n*) for each experimental group/condition, given as a discrete number and unit of measurement
☐	☒	A statement on whether measurements were taken from distinct samples or whether the same sample was measured repeatedly
☐	☒	The statistical test(s) used AND whether they are one- or two-sided<br>*Only common tests should be described solely by name; describe more complex techniques in the Methods section.*
☐	☒	A description of all covariates tested
☐	☒	A description of any assumptions or corrections, such as tests of normality and adjustment for multiple comparisons
☐	☒	A full description of the statistical parameters including central tendency (e.g. means) or other basic estimates (e.g. regression coefficient) AND variation (e.g. standard deviation) or associated estimates of uncertainty (e.g. confidence intervals)
☐	☒	For null hypothesis testing, the test statistic (e.g. *F*, *t*, *r*) with confidence intervals, effect sizes, degrees of freedom and *P* value noted<br>*Give P values as exact values whenever suitable.*
☒	☐	For Bayesian analysis, information on the choice of priors and Markov chain Monte Carlo settings
☒	☐	For hierarchical and complex designs, identification of the appropriate level for tests and full reporting of outcomes
☒	☐	Estimates of effect sizes (e.g. Cohen's *d*, Pearson's *r*), indicating how they were calculated

Our web collection on statistics for biologists contains articles on many of the points above.

Software and code

Policy information about availability of computer code

Data collection	Omega MARS v3.32, Attune NxT Software v3.1.2.0, Image Lab v6.0.0
Data analysis	Omega MARS v3.32, Microsoft Excel 2013, GraphPad Prism v8.1.2, Attune NxT Software v3.1.2.0, FlowJo v7.6.1, Image Lab v6.0.0

For manuscripts utilizing custom algorithms or software that are central to the research but not yet described in published literature, software must be made available to editors and reviewers. We strongly encourage code deposition in a community repository (e.g. GitHub). See the Nature Research guidelines for submitting code & software for further information.

Data

Policy information about availability of data

All manuscripts must include a data availability statement. This statement should provide the following information, where applicable:

- Accession codes, unique identifiers, or web links for publicly available datasets
- A list of figures that have associated raw data
- A description of any restrictions on data availability

All data and plasmids supporting the findings are available from the corresponding author upon reasonable request. Plasmids of the six stimuli-responsive genetic circuits, all the single-layer DNA sponges, and representative dual-layer sponges are available from Addgene (ID in brackets): pXW117Ptet2-gfp (160818), pXW101Plux2-gfp (160819), pXW101Plux2-33Ptet2-gfp (160820), pXW101Plux2-32Ptet2-gfp (160821), pXW109PmerT-Pecf11-gfp (160822), pXW101Plux2-Pecf11-gfp (160823), pXWStetO (160824), pXWS10tetO (160825), pXWS20tetO (160826), pXWS40tetO (160827), pXWS80tetO (160828), pXWS160tetO (160829), pXWS320tetO (160830), pXWSPtet2 (160831), pXWS5Ptet2 (160832), pXWS10Ptet2 (160833), pXWS20Ptet2 (160834), pXWS40Ptet2 (160835), pXWSLBS (160836), pXWS10LBS (160837), pXWS20LBS (160838), pXWS40LBS (160839), pXWS80LBS (160840), pXWSPecf11 (160841), pXWS10Pecf11 (160842), pXWS20Pecf11 (160843), pXWS40Pecf11 (160844), PXWS80LBS40tetO (160845), PXWS80LBS320tetO (160846), PXWS20LBS20Pecf11 (160847), PXWS80LBS40Pecf11 (160848). Source data are provided with this paper as a Source Data file underlying Figs. 2b–e,g,h, 3b–d,f–k, 4b–e, 5b–i, 6b,d,e, Supplementary Figs. 11a and 12a.

Field-specific reporting

Please select the one below that is the best fit for your research. If you are not sure, read the appropriate sections before making your selection.

- [x] Life sciences
- [ ] Behavioural & social sciences
- [ ] Ecological, evolutionary & environmental sciences

For a reference copy of the document with all sections, see nature.com/documents/nr-reporting-summary-flat.pdf

Life sciences study design

All studies must disclose on these points even when the disclosure is negative.

Sample size	Sample sizes (= number of replicate experiments) were chosen according to common practices in the field and were such that standard deviations were small enough to allow determination of significant effects and trends. In all cases, three independent replicates (minimum for statistical analysis like error bars and significance tests) were carried out for each experiment.
Data exclusions	No data were excluded from the analyses.
Replication	All experiments were performed in three replicates. All experiments were biological replicates. All attempts at replication were successful.
Randomization	For each experimental group, each biological replicate of a bacterial culture was inoculated from a single colony, which was randomly chosen from an agar plate.
Blinding	No blinding was involved as it was not relevant to this study because our data are not based on qualitative scoring metrics. No animal or human research participants were utilized and all samples were processed in parallel. Blinding during group allocation is also irrelevant in our study because samples of bacterial cultures that were split into different conditions were random samplings and there is no control over which cells will be selected and thus there is no bias during group allocation.

Reporting for specific materials, systems and methods

We require information from authors about some types of materials, experimental systems and methods used in many studies. Here, indicate whether each material, system or method listed is relevant to your study. If you are not sure if a list item applies to your research, read the appropriate section before selecting a response.

Materials & experimental systems

n/a	Involved in the study	
x		Antibodies
x		Eukaryotic cell lines
x		Palaeontology and archaeology
x		Animals and other organisms
x		Human research participants
x		Clinical data
x		Dual use research of concern

Methods

n/a	Involved in the study	
x		ChIP-seq
	x	Flow cytometry
x		MRI-based neuroimaging

Flow Cytometry

Plots

Confirm that:

- [ ] The axis labels state the marker and fluorochrome used (e.g. CD4-FITC).
- [x] The axis scales are clearly visible. Include numbers along axes only for bottom left plot of group (a 'group' is an analysis of identical markers).
- [x] All plots are contour plots with outliers or pseudocolor plots.
- [x] A numerical value for number of cells or percentage (with statistics) is provided.

Methodology

Sample preparation	Bacterial cells from the 96-well plate culture were transferred and diluted 1 : 100 to another U-bottom 96-well plate with PBS (1×, with 2 mg/ml Kanamycin to stop translation). The transferred plate was incubated at 4°C for at least 1 h before the flow cytometry assay.
Instrument	Attune NxT Flow Cytometer and Attune NxT Autosampler.

Software	Attune NxT Software v3.1.2.0, Microsoft Excel 2013, GraphPad Prism v8.1.2, FlowJo v7.6.1
Cell population abundance	Bacterial cells were gated based on the cell's size and complexity during the flow cytometry assay. 10,000 of gated cells were collected for analyses.
Gating strategy	Live bacterial cells were gated by FCS and SSC. Green fluorescence was detected by BL1 channel (with 488 nm excitation and 530 nm emission). No boundaries between 'positive' and 'negative' cell populations were defined.

☒ Tick this box to confirm that a figure exemplifying the gating strategy is provided in the Supplementary Information.
